# Comparison of Severe Maternal Morbidities Associated With Delivery During Periods of Circulation of Specific SARS-CoV-2 Variants

**DOI:** 10.1001/jamanetworkopen.2022.26436

**Published:** 2022-08-12

**Authors:** Maria Mupanomunda, Mohamad G. Fakih, Collin Miller, Allison Ottenbacher, Angela L. Winegar, Phillip Roberts, Moyo Kimathi, John G. Gianopoulos, Alison G. Cahill, Joseph G. Cacchione, Richard I. Fogel, Thomas A. Aloia, Frederick A. Masoudi

**Affiliations:** 1 Ascension Health, St Louis, Missouri; 2 Dell Medical School, University of Texas, Austin

## Abstract

**Importance:**

Infection with SARS-CoV-2, which causes COVID-19, is associated with adverse maternal outcomes. While it is known that severity of COVID-19 varies by viral strain, the extent to which this variation is reflected in adverse maternal outcomes, including nonpulmonary maternal outcomes, is not well characterized.

**Objective:**

To evaluate the associations of SARS-CoV-2 infection with severe maternal morbidities (SMM) in pregnant patients delivering during 4 pandemic periods characterized by predominant viral strains.

**Design, Setting, and Participants:**

This retrospective cohort study included patients delivering in a multicenter, geographically diverse US health system between March 2020 and January 2022. Individuals with SARS-CoV-2 infection were propensity-matched with as many as 4 individuals without evidence of infection based on demographic and clinical variables during 4 time periods based on the dominant strain of SARS-CoV-2: March to December 2020 (wild type); January to June 2021 (Alpha [B.1.1.7]); July to November 2021 (Delta [B.1.617.2]); and December 2021 to January 2022 (Omicron [B.1.1.529]). Data were analyzed from October 2021 to June 2022.

**Exposures:**

Positive SARS-CoV-2 nucleic acid amplification test result during the delivery encounter.

**Main Outcomes and Measures:**

The primary outcome was any SMM event, as defined by the US Centers for Disease Control and Prevention, during hospitalization for delivery. Secondary outcomes were number of SMM, respiratory SMM, nonrespiratory SMM, and nontransfusion SMM events.

**Results:**

Over all time periods, there were 3129 patients with SARS-CoV-2, with a median (IQR) age of 29.1 (24.6-33.2) years. They were propensity matched with a total of 12 504 patients without SARS-CoV-2, with a median (IQR) age of 29.2 (24.7-33.2) years. Patients with SARS-CoV-2 infection had significantly higher rates of SMM events than those without in all time periods, except during Omicron. While the risk of any SMM associated with SARS-CoV-2 infection was increased for the wild-type strain (odds ratio [OR], 2.74 [95% CI, 1.85-4.03]) and Alpha variant (OR, 2.57 [95% CI, 1.69-4.01]), the risk during the Delta period was higher (OR, 7.69 [95% CI, 5.19-11.54]; *P* for trend < .001). The findings were similar for respiratory complications, nonrespiratory complications, and nontransfusion outcomes. For example, the risk of nonrespiratory SMM events for patients with vs without SARS-CoV-2 infection were similar for the wild-type strain (OR, 2.16 [95% CI, 1.40-3.27]) and Alpha variant (OR, 1.96 [95% CI, 1.20-3.12]), highest for the Delta variant (OR, 4.65 [95% CI, 2.97-7.29]), and not significantly higher in the Omicron period (OR, 1.21 [95% CI, 0.67-2.08]; *P* for trend < .001).

**Conclusions and Relevance:**

This cohort study found that the SARS-CoV-2 Delta variant was associated with higher rates of SMM events compared with other strains. Given the potential of new strains, these findings underscore the importance of preventive measures.

## Key Points

**Question:**

Does the association between SARS-CoV-2 infection and severe maternal morbidity (SMM), including nonrespiratory complications, vary by viral strain?

**Findings:**

In this retrospective cohort study of 3129 patients with SARS-CoV-2 infection and 12 504 patients without infection giving birth in a large US health system between March 2020 and January 2022, the risk of SMM associated with SARS-CoV-2 infection was significantly higher during the phase of the pandemic when the Delta variant was predominant (July 2021-November 2021). This association was also noted specifically for both respiratory and nonrespiratory SMM.

**Meaning:**

These findings highlight the importance of the prevention of SARS-CoV-2 infection in pregnant individuals and the consideration of infection as a risk factor for adverse peripartum maternal outcomes.

## Teaser

This cohort study evaluates the association of SARS-CoV-2 infection and severe maternal morbidities in patients giving birth during 4 pandemic periods characterized by predominant viral strains.

## Introduction

Between January 2020 and February 2022, more than 170 000 pregnant people in the US were infected by SARS-CoV-2 and 29 000 pregnant people were hospitalized with COVID-19.^1^ Pregnancy can be associated with a hypercoagulable state, altered immune function, and increased susceptibility to hypoxemia, which could potentially worsen maternal outcomes with SARS-CoV-2 infection.^2,3^ The findings of studies from early in the COVID-19 pandemic on the impact of SARS-CoV-2 infection on pregnancy outcomes have not been definitive, perhaps in part owing to limited sample sizes. Some studies found higher rates of maternal complications, such as caesarian and preterm deliveries,^4,5^ while others did not identify an increase in maternal risk.^6,7^ As the pandemic evolved, with greater spread and the emergence of SARS-CoV-2 variants, more studies reported an association between SARS-CoV-2 infection and higher rates of poor maternal outcomes, including eclampsia, preeclampsia, HELLP syndrome (hemolysis, elevated liver enzymes and low platelets), sepsis, intensive care unit admission, preterm birth and low birth weight, and, more recently, maternal death or serious morbidity related to hypertensive disorders of pregnancy, postpartum hemorrhage, or infection other than SARS-CoV-2.^8,9,10,11^

The pandemic has been characterized by several waves defined by viral strains that are responsible for the preponderance of infections. In July 2021, the Delta (B.1.617.2) variant of SARS-CoV-2 became the predominant variant in the US.^12^ One study^13^ from a single hospital found higher COVID-19 caseloads and severity of illness among pregnant people during the Delta surge compared with previous surges. However, this study did not assess adverse maternal outcomes around the time of delivery. Another early study^14^ at 1 institution presented findings suggesting serious morbidity and adverse perinatal outcomes associated with the Delta variant in pregnancy. In December 2021, Omicron became the predominant strain in the US, and although this strain continued to be responsible for hospitalizations and deaths, disease severity was generally less than the Delta strain.^15^ Maternal outcomes for people infected with the Omicron variant are not well characterized.

The primary objective of our study is to evaluate the association between SARS-CoV-2 infection and severe maternal morbidities (SMM), as defined by the US Centers for Disease Control and Prevention (CDC), in pregnant patients during pandemic periods when the wild-type strain or Alpha (B.1.1.7), Delta, or Omicron (B.1.1.529) variants of SARS-CoV-2 predominated. The CDC defines SMM as unexpected outcomes of labor and delivery, consisting of 21 health indicators, that result in significant short- or long-term consequences to an individual’s health.^16^ To address this question, we studied pregnant patients delivering in a geographically diverse multistate US health system between March 2020 and January 2022.

## Methods

This cohort study was deemed exempt from review and informed consent by the Ascension Seton Institutional Review Board under 45 CFR 46.104 of the Common Rule. This study is reported following the Strengthening the Reporting of Observational Studies in Epidemiology (STROBE) reporting guideline.

### Setting

This is a retrospective study of pregnant patients who delivered in any of 32 hospitals in a single health system from March 2020 through January 2022. These hospitals were located in 8 US states: Alabama, Florida, Indiana, Maryland, Michigan, New York, Tennessee, and Texas. We constructed 4 time periods based on the dominant strain of SARS-CoV-2 in the US: March 2020 to December 2020 (wild-type strain), January 2021 to June 2021 (Alpha variant), July 2021 to November 2021 (Delta variant), and December 2021 to January 2022 (Omicron variant).^12^

### Population

All patients who delivered during the study period in any of 32 hospitals within the health system were candidates for inclusion. Individuals were identified to have a SARS-CoV-2 infection if they had a positive result using polymerase chain reaction nucleic acid amplification testing for SARS-CoV-2 infection during the encounter for delivery. Individuals with a negative test result or for whom test results were not available were categorized as controls. Early in the pandemic, owing to limited testing capacity, only patients with symptoms that would qualify for a person under investigation, including fever, cough, dyspnea, diarrhea, or a close contact with someone with SARS-CoV-2 infection, were tested at admission. Universal testing for all pregnant patients admitted for delivery was initiated in May 2020, during the wild-type strain period. Because of this variation in testing practices across pandemic periods, outcomes were compared in sensitivity analyses between those with confirmed SARS-CoV2 infection and those with a documented negative test result for SARS-CoV2 (ie, excluding those without test results).

### Data Sources

SMM outcomes, SARS-CoV-2 infection status, and other patient information were derived from a combination of health system data, including acute care claims data for inpatient encounters and standardized data elements from electronic health records. Race and ethnicity were self-reported for most patients, but the source could not be confirmed for all reviewed records. Race was categorized as Black, White, and other, which included all races other than White or Black, as well as those for whom race was unknown or for whom documentation of race was declined. If race was unknown but ethnicity was Hispanic or Latino, the person was classified as Hispanic or Latino. If ethnicity was unknown, non-Hispanic or Latino ethnicity was assumed. Race and ethnicity were assessed because of potential differential associations with maternal outcomes independent of SARS-CoV-2 infection. Missing demographic data were not altered and are reported as unknown. For each individual SMM, a null value was considered to be a nonevent and not counted in the totals.

### Outcomes

The primary outcome was any SMM event occurring during the hospitalization for delivery. These events are defined by the CDC as including unexpected outcomes of labor and delivery that result in significant short- or long-term consequences to an individual’s health.^16^ To identify delivery hospitalizations with SMM, the CDC uses administrative hospital discharge data and *International Statistical Classification of Diseases and Related Health Problems, Tenth Revision (ICD-10)* diagnosis and procedure codes. The CDC’s 2019 list of SMM includes 21 health indicators (ie, acute myocardial infarction, aneurysm, acute kidney failure, adult respiratory distress syndrome [ARDS], amniotic fluid embolism, cardiac arrest or ventricular fibrillation, conversion of cardiac rhythm, disseminated intravascular coagulation, eclampsia, heart failure or arrest during operation or procedure, puerperal cerebrovascular disorders, pulmonary edema or acute heart failure, severe anesthesia complications, sepsis, shock, sickle cell disease with crisis, air and thrombotic embolism, blood products transfusion, hysterectomy, temporary tracheostomy, and ventilation).^16^ Secondary outcomes included the number of SMM events, any respiratory SMM (ie, ARDS, mechanical ventilation, temporary tracheostomy), nonrespiratory SMM events, and nontransfusion SMM events.

### Statistical Analysis

Categorical variables were summarized as frequencies and percentages, and were compared using χ^2^ or Fisher exact tests. Age was treated as a continuous variable, presented as median and IQR, and compared using the Wilcoxon rank-sum test. A multivariable logistic regression model with demographic and clinical variables was performed to estimate a propensity score with which individuals with SARS-CoV-2 infection were matched to those without infection. Nearest-neighbor matching was performed with each case matched to a maximum of 4 controls using a caliper of 0.2. Propensity score modeling and matching were performed independently within each of the 4 time frames. Standardized mean differences quantified the quality of the match, with differences less than 10% representing acceptable balance on covariates. Matched data were used to compare the rates of SMM events between those with and without SARS-CoV-2 infection for each variant time frame. A 2-sided *P* < .05 was designated as statistically significant. All statistical analyses were performed in R statistical software version 4.0.2 (R Project for Statistical Computing). Data were analyzed from October 2021 to June 2022.

## Results

### Population

Overall, 3129 patients with SARS-CoV-2 infection, with a median (IQR) age of 29.1 (24.6-33.2) years and 98 325 patients without documented infection delivered at 32 facilities between March 2020 and January 2022 (eTable 1 in the Supplement). Demographic characteristics that differed significantly between patients with and without documented SARS-CoV-2 infection before propensity score matching in 1 or more time periods include insurance type, Hispanic ethnicity, race, and age (eTable 1 in the Supplement). Comorbidities that differed significantly between those with and without SARS-CoV-2 infection in 1 or more time frames included obesity, asthma, gestational diabetes, and anemia. In all 4 time periods, patients with SARS-CoV-2 were more likely to have a preterm delivery (<37 weeks gestation) than those without SARS-CoV-2 infection (eTable 1 in the Supplement).

In the final analysis there were 3129 patients with SARS-CoV-2 infection matched to 12 504 patients (median [IQR] age, 29.2 [24.7-33.2] years) without evidence of infection, with 978 cases and 3906 controls in the wild-type strain period; 744 cases and 2974 controls in the Alpha period; 681 cases and 2724 controls in the Delta period, and 726 cases and 2900 controls in the Omicron period (Table 1). Among patients with SARS-CoV-2 infection, 21.1% were Black, 24.2% were Hispanic or Latino, 75.8% were not Hispanic or Latino, and 65.3% were White, while among those without SARS-CoV-2 infection, 20.3% were Black, 24.8% were Hispanic or Latino, 75.2% were not Hispanic or Latino, and 66.4% were White. For the 4 time periods, standardized mean differences were less than 10% on all covariates for propensity-matched patients (eFigure in the Supplement).

**Table 1.** Patient Characteristics by SARS-CoV-2 Status and Variant in Propensity Matched Populations

Characteristic	SARS-CoV-2 test result by period, No. (%)							
	Wild-type^a^		Alpha^b^		Delta^c^		Omicron^d^	
	Positive (n = 978)^e^	Not positive (n = 3906)	Positive (n = 744)^f^	Not positive (n = 2974)	Positive (n = 681)	Not positive (n = 2724)	Positive (n = 726)^g^	Not positive (n = 2900)
Age, median (IQR), y	29.5 (24.8-33.5)	29.6 (24.8-33.7)	29.0 (24.4-33.5)	28.8 (24.6-33.3)	29.1 (25.0-33.1)	29.2 (25.1-33.0)	28.6 (24.5-32.8)	28.7 (24.4-32.8)
Race								
White	618 (63.2)	2513 (64.3)	499 (67.1)	2038 (68.5)	460 (67.5)	1889 (69.3)	467 (64.3)	1863 (64.2)
Black	209 (21.3)	789 (20.2)	155 (20.8)	600 (20.2)	142 (20.9)	542 (19.9)	154 (21.2)	608 (21.0)
Other^h^	111 (11.3)	447 (11.4)	62 (8.3)	240 (8.1)	66 (9.7)	246 (9.0)	84 (11.6)	348 (12.0)
Unknown or declined	40 (4.1)	157 (4.0)	28 (3.8)	96 (3.2)	13 (1.9)	47 (1.7)	21 (2.9)	81 (2.8)
Ethnicity								
Hispanic or Latino	323 (33.0)	1303 (33.4)	156 (21.0)	611 (20.5)	140 (20.6)	543 (19.9)	160 (22.0)	646 (22.3)
Not Hispanic or Latino or unknown	655 (67.0)	2603 (66.6)	588 (79.0)	2363 (79.5)	541 (79.4)	2181 (80.1)	566 (78.0)	2254 (77.7)
Payer								
Private	412 (42.1)	1657 (424)	380 (51.1)	1550 (52.1)	351 (51.5)	1457 (53.5)	349 (48.1)	1409 (48.6)
Public	539 (55.1)	2153 (55.1)	353 (47.4)	1369 (46.0)	316 (46.4)	1214 (44.6)	361 (49.7)	1422 (49.0)
Uninsured or self-pay	26 (2.7)	95 (2.4)	11 (1.5)	55 (1.8)	11 (1.6)	47 (1.7)	15 (2.1)	66 (2.3)
Other or unknown	1 (0.1)	1 (<0.1)	0	0	3 (0.4)	6 (0.2)	1 (0.1)	3 (0.1)
Obesity	158 (16.2)	591 (15)	131 (17.6)	525 (17.7)	91 (13.4)	330 (12.1)	99 (14)	380 (13.1)
Asthma	72 (7.4)	301 (7.7)	51 (6.9)	220 (7.4)	45 (6.6)	173 (6.4)	52 (7.2)	198 (6.8)
Diabetes	143 (14.6)	565 (15.1)	95 (12.8)	397 (13.3)	71 (10.4)	291 (10.7)	75 (10.3)	297 (10.2)
Anemia	96 (9.8)	377 (9.7)	69 (9.3)	251 (8.4)	86 (12.6)	336 (12.3)	94 (12.9)	375 (12.9)
Preterm delivery <37 weeks gestation	137 (14.0)	535 (13.7)	106 (14.2)	442 (14.9)	138 (20.3)	573 (21.0)	109 (15.0)	438 (15.1)

^a^ Defined as March to December 2020.

^b^ Defined as January to June 2021.

^c^ Defined as July to November 2021.

^d^ Defined as December 2021 to January 2022.

^e^ One individual with a positive test result had only 1 match, 1 individual had only 2 matches, and 1 individual had 3 matches.

^f^ One individual with a positive test result had only 2 matches.

^g^ Four individuals with positive test results had only 3 matches.

^h^ Includes individuals with American Indian or Alaska Native, Asian, not reported, or missing race.

### All SMM

Patients with SARS-CoV-2 infection had significantly higher rates of any SMM event than those without infection in all time periods, except Omicron (Table 2, Figure, A). Rates of individual SMM events during each pandemic wave period are presented in eTable 2 in the Supplement. While the risk was similarly increased for the wild-type strain (44 patients [4.5%] vs 66 patients [1.7%]; propensity-matched odds ratio [OR], 2.74 [95% CI, 1.85-4.03]) and Alpha variant (35 patients [4.7%] vs 55 patients [1.8%]; OR, 2.62 [95% CI, 1.69-4.01]) periods, the risk for the Delta period was higher (70 patients [10.3%] vs 55 patients [1.5%]; OR, 7.69 [95% CI, 5.19-11.54]; *P* for trend < .001). The risk for any SMM associated with the Omicron variant was not statistically significant (OR, 1.60 [95% CI, 0.94-2.63]). Notably, the risk of SMM in the group without evidence of SARS-CoV-2 infection was consistent among the time periods.

**Table 2.** Severe Maternal Morbidity Outcomes by SARS-CoV-2 Status and Variant Type

SMM	SARS-CoV-2 test result, No. (%)							
	Wild-type^a^		Alpha^b^		Delta^c^		Omicron^d^	
	Positive (n = 978)^e^	Not positive (n = 3906)	Positive (n = 744)^f^	Not positive (n = 2974)	Positive (n = 681)	Not positive (n = 2724)	Positive (n = 726)^g^	Not positive (n = 2900)
Any	44 (4.5)	66 (1.7)	35 (4.7)	55 (1.8)	70 (10.3)	40 (1.5)	21 (2.9)	53 (1.8)
Respiratory	18 (1.8)	6 (0.2)	19 (2.6)	5 (0.2)	46 (6.8)	5 (0.2)	7 (1.0)	4 (0.1)
Non-respiratory	34 (3.5)	64 (1.6)	26 (3.5)	54 (1.8)	42 (6.2)	38 (1.4)	16 (2.2)	53 (1.8)
Non-transfusion	33 (3.4)	32 (0.8)	28 (3.8)	22 (0.7)	58 (8.5)	17 (0.6)	13 (1.8)	18 (0.6)

Abbreviation: SMM, severe maternal morbidity.

^a^ Defined as March to December 2020.

^b^ Defined as January to June 2021.

^c^ Defined as July to November 2021.

^d^ Defined as December 2021 to January 2022.

^e^ One individual with a positive test result had only 1 match, 1 individual had only 2 matches, and 1 individual had 3 matches.

^f^ One individual with a positive test result had only 2 matches.

^g^ Four individuals with positive test results had only 3 matches.

**Figure.**
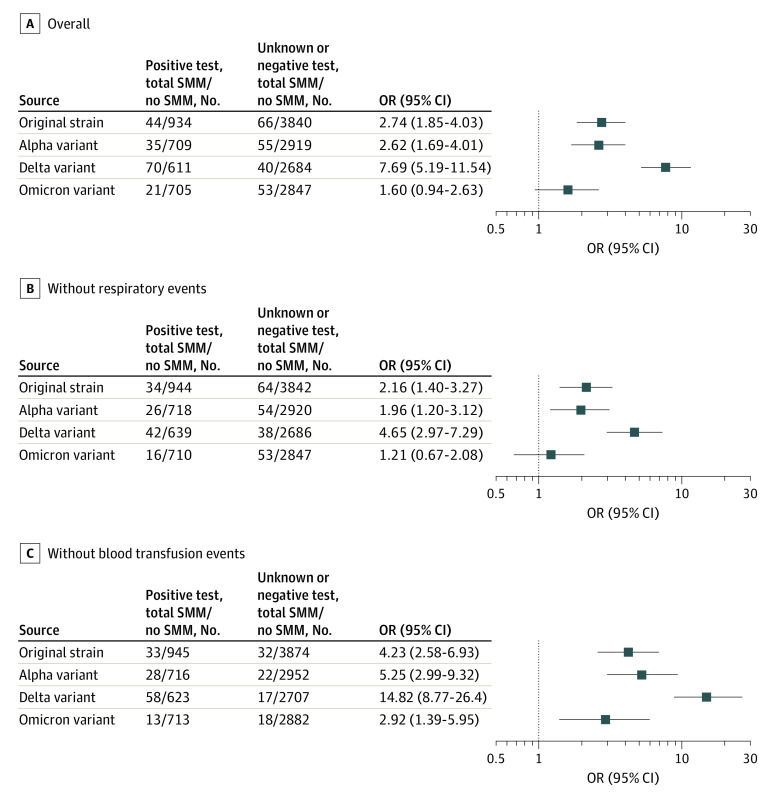
Propensity-Matched Odds of Severe Maternal Morbidities (SMM) Associated With SARS-CoV-2 Infection OR indicates odds ratio.

### Secondary Outcomes

In an analysis restricted only to respiratory SMM, patients with SARS-CoV-2 infection had significantly higher observed rates of respiratory SMM for all variants (Table 2). Consistent with the primary outcome, the risk of respiratory SMM events for patients with and without SARS-CoV-2 infection were similar for the wild-type strain (OR, 12.79 [95% CI, 5.09-33.69]) and Alpha variant (OR, 15.56 [95% CI, 6.23-47.06]), highest for the Delta variant (OR, 28.14 [95% CI, 17.15-113.99]), and lowest for the Omicron variant (OR, 7.05 [95% CI, 2.12-26.97]; *P* for trend < .001). In an analysis restricted only to nonrespiratory SMM, patients with SARS-CoV-2 infection had significantly higher observed rates of nonrespiratory SMM for all variants, except Omicron (Table 2). The odds for a nonrespiratory SMM associated with SARS-CoV-2 infection were similar for the wild-type strain (OR, 2.16 [95% CI, 1.40-3.27]) and Alpha variant (OR, 1.96 [95% CI, 1.20-3.12]), highest for the Delta variant (OR, 4.65 [95% CI, 2.97-7.29]), and not significantly higher in the Omicron period (OR, 1.21 [95% CI, 0.67-2.08]; *P* for trend < .001) (Figure, B). In an analysis restricted only to nontransfusion SMM, consistent with analysis of the primary outcome, the risk of adverse events was higher among patients with SARS-CoV-2 infection for the wild-type strain (OR, 4.23 [95% CI, 2.58-6.93]) and Alpha variant (OR, 5.25 [95% CI, 2.99-9.32]), highest for the Delta variant (OR, 14.82 [95% CI, 8.77-26.4], and lower but still significant for the Omicron variant (OR, 2.92 [95% CI, 1.39-5.95], *P* for trend < .001) (Figure, C).

### Sensitivity Analysis

In secondary analyses, the control group was restricted only to patients with a documented negative test result for SARS-CoV-2. The characteristics of the propensity-matched population were similar to that of the primary analysis (eTable 3 in the Supplement). The associations of SARS-CoV-2 infection with the primary outcome of all SMM and the secondary outcomes by SMM type were also similar (eTable 4 in the Supplement).

## Discussion

In this cohort study in a demographically and geographically diverse population in a US health system, we found variable associations of SARS-CoV-2 infection with CDC-defined rates of SMM during different pandemic periods. SARS-CoV-2 infection at the time of delivery was significantly associated with SMM in all but the Omicron period; the magnitude of the associations was most pronounced during the time period when the Delta variant was predominant. This pattern was consistent even for nonrespiratory complications, for nontransfusion complications, and in sensitivity analyses including only individuals with documented results for SARS-CoV-2 testing. Our findings corroborate those of a study by Metz et al^11^ of pregnant or postpartum patients reporting that SARS-CoV-2 was associated with increased risk for a composite outcome of maternal mortality or serious morbidity from obstetric complications. Our study is complementary in that it compares 4 pandemic periods with different strain dominance; focuses only on patients during the delivery episode, the time most biologically plausible to influence the outcomes measured; demonstrates associations during later pandemic periods when universal screening had been instituted; assesses nonrespiratory adverse outcomes; and includes a broad population and spectrum of obstetrics units.

We found that the risk associated with COVID-19, while particularly high for respiratory complications, was also relevant to nonrespiratory complications around the time of delivery. Nonrespiratory complications were also more pronounced with the Delta variant compared with the Alpha variant or the wild-type SARS-CoV-2 strain. The nonrespiratory SMM associated with COVID-19 were driven, in part, by higher than expected rates of blood product transfusion during the Delta variant period. This finding supports prior reports suggesting higher rates of nonpulmonary complications (eg, postpartum hemorrhage and blood transfusions) in pregnant patients with SARS-CoV-2 infection during gestation than in the general population.^17^ Our findings add to this work by comparing rates of nonrespiratory complications specifically around the time of delivery and the differential associations of SARS-CoV-2 strains. Additionally, our findings could help inform maternal risk stratification in terms of hemorrhage risk and anticipation of resources needed. While in general, blood product transfusions account for the largest proportion of SMM,^18^ we cannot identify the mechanism for the higher risk of blood transfusion in our study, a finding that merits further investigation.

Our study adds to previous work by examining and demonstrating the risk of adverse maternal outcomes specifically at the time of labor and delivery and comparison associated with 4 strains of SARS-CoV-2. Previous studies examined the outcomes of COVID-19 in pregnant patients throughout pregnancy.^13,19^ With the potential emergence of future SARS-CoV-2 variants and the magnitude of the risk for adverse maternal outcomes associated with SARS-CoV-2 infection and particularly with the Delta variant, our study underscores the critical importance of promoting prevention strategies to reduce risk to pregnant people at the time of delivery. Several recent studies, as well as recommendations from the CDC and professional organizations, including the American College of Obstetricians and Gynecologists, the Society of Maternal-Fetal Medicine, and the American Academy of Pediatrics, all support the promotion of COVID-19 vaccination in pregnant people and people of childbearing age to reduce risk of adverse outcomes.^20,21,22,23,24^ Despite these recommendations, as well as studies showing vaccines are safe in pregnancy, COVID-19 vaccine uptake remains low among pregnant people and people of childbearing age.^25,26,27^ Furthermore, misinformation regarding the vaccine is widespread, including among people who are pregnant or who are planning to get pregnant.^28^ Our findings underscore the importance of promoting vaccination acceptance and combating misinformation together with other prevention strategies in pregnant individuals. In addition, early diagnosis of infection and prompt therapeutic intervention for SARS-CoV-2 infection with antivirals and monoclonal antibodies may reduce the frequency and severity of maternal morbidities.^21,29,30,31^

### Limitations

Although this is one of the largest studies investigating the associations of different strains of SAR-CoV2 with SMM in a geographically and demographically diverse population during the pandemic, the results of this study should be interpreted in the context of several limitations. First, we were unable to account for maternal vaccination status owing to inadequate data availability. While this is not relevant to the original period and would only have a modest impact on the Alpha variant period, it would have a greater impact on later periods. In any event, because maternal testing was universal in the later periods, and because vaccination is generally associated with less severe SARS-CoV-2 infection, the differences in outcomes in the later periods relative to the original period associated with infection would, if anything, be biased toward the null. Second, not all patients early in the wild-type strain period were tested for SARS-CoV-2 infection. In periods when universal testing was used, we were unable to differentiate between incidentally discovered SARS-CoV2 infection vs symptomatic COVID-19. In principal analysis, patients who were not tested were assumed to be negative. This raises the question of potential surveillance bias early in the wild-type strain period, when patients with symptoms were more likely to be tested than those without symptoms. However, the results remained consistent even when we removed patients who were not tested for SARS-CoV-2 from the analysis. Third, we were unable to identify patients who had experienced a SARS-CoV-2 infection during their pregnancy and had recovered to the point when testing would not identify an active infection. Thus, it is possible that some of the adverse outcomes in patients without a positive test result could have been attributable to an earlier infection. Again, this issue would bias the results of the study toward the null. Fourth, we did not have sequencing data to identify the strain in individuals with SARS-CoV-2 infection. We used time period as a proxy for the dominant strain of SARS-CoV-2. However, epidemiology data suggest that the selected strains (wild-type, Alpha, Delta, and Omicron) were dominant and accounted for more than 70% of infections during each of the 4 time periods used. Fifth, because the study is limited to hospitals within a single health system, the results may not reflect outcomes in the broader US population. However, the population of sites and individuals is geographically and demographically diverse.

## Conclusions

In this cohort study in a large US health system, infection with SARS-CoV-2 at the time of delivery throughout the pandemic was associated with higher rates of SMM; however, this association was particularly strong with the Delta strain. This variable association with viral strain was present both for respiratory and nonrespiratory complications, as well as complications not related to blood transfusion. These findings highlight the importance of SARS-CoV-2 prevention in pregnant patients and consideration of infection as an indicator of high risk in the peripartum period.

## Notes

Supplement. **eTable 1.** Population Characteristics in the Unmatched Population **eFigure.** Standardized Mean Differences Between Groups With and Without SARS-CoV-2 Infection Before and After Propensity Matching **eTable 2.** Distribution of Individual SMM, by Variant **eTable 3.** Baseline Characteristics Comparing Populations With Documented SARS-CoV-2 Testing After Propensity Matching **eTable 4.** Association of SARS-CoV-2 Infection With Any SMM, Nonrespiratory SMM, Respiratory SMM, and Nontransfusion SMM
