# Supplementary material for: Comparison of Severe Maternal Morbidities Associated With Delivery During Periods of Circulation of Specific SARS-CoV-2 Variants
Source: JAMA Netw Open. 2022 Aug 12;5(8):e2226436. doi: 10.1001/jamanetworkopen.2022.26436 (PMC9375165; doi:10.1001/jamanetworkopen.2022.26436)
Supplement: Supplement. — eTable 1. Population Characteristics in the Unmatched Population eFigure. Standardized Mean Differences Between Groups With and Without SARS-CoV-2 Infection Before and After Propensity Matching eTable 2. Distribution of Individual SMM, by Variant eTable 3. Baseline Characteristics Comparing Populations With Documented SARS-CoV-2 Testing After Propensity Matching eTable 4. Association of SARS-CoV-2 Infection With Any SMM, Nonrespiratory SMM, Respiratory SMM, and Nontransfusion SMM [file jamanetwopen-e2226436-s001.pdf]

# Supplemental Online Content

Mupanomunda M, Fakih MG, Miller C, et al. Comparison of severe maternal morbidities associated with delivery during periods of circulation of specific SARS-CoV-2 variants. *JAMA Netw Open*. 2022;5(8):e2226436. doi:10.1001/jamanetworkopen.2022.26436

**eTable 1.** Population Characteristics in the Unmatched Population

**eFigure.** Standardized Mean Differences Between Groups With and Without SARS-CoV-2 Infection Before and After Propensity Matching

**eTable 2.** Distribution of Individual SMM, by Variant

**eTable 3.** Baseline Characteristics Comparing Populations With Documented SARS-CoV-2 Testing After Propensity Matching

**eTable 4.** Association of SARS-CoV-2 Infection With Any SMM, Nonrespiratory SMM, Respiratory SMM, and Nontransfusion SMM

This supplemental material has been provided by the authors to give readers additional information about their work.

© 2022 Mupanomunda M et al. *JAMA Network Open.*

# eTable 1. Population Characteristics in the Unmatched Population

| | Original SARS-CoV-2 Strain (03/2020-12/2020) | | | Alpha SARS-CoV-2 Variant (01/2021-06/2021) | | | Delta SARS-CoV-2 Variant (07/2021-11/2021) | | | Omicron SARS-CoV Variant (12/2021-01/2022) | | | Group Total | | | Total |
|---|---|---|---|---|---|---|---|---|---|---|---|---|---|---|---|---|
| **Characteristic** | **Unknown/Test-Negative N = 43,033** | **Test-Positive N = 978** | **p-value** | **Unknown/Test-Negative N = 25,168** | **Test-Positive N = 744** | **p-value** | **Unknown/Test-Negative N = 22,324** | **Test-Positive N = 681** | **p-value** | **Unknown/Test-Negative N = 7,800** | **Test-Positive N = 726** | **p-value** | **Unknown/Test-Negative N = 98,325** | **Test-Positive N = 3,129** | **p-value** | **N = 101,454** |
| **Age** | 29.6 (25.4, 33.6) | 29.5 (24.8, 33.5) | 0.072 | 29.7 (25.5, 33.6) | 29.0 (24.4, 33.5) | 0.015 | 29.9 (25.7, 33.8) | 29.1 (25.0, 33.1) | 0.006 | 29.8 (25.4, 33.8) | 28.6 (24.5, 32.8) | <0.001 | 29.7 (25.5, 33.7) | 29.1 (24.6, 33.2) | <0.001 | 29.7 (25.4, 33.6) |
| **Race** | | | <0.001 | | | 0.005 | | | 0.06 | | | 0.2 | | | <0.001 | |
| White | 29,424 (68%) | 618 (63%) | | 17,396 (69%) | 499 (67%) | | 15,171 (68%) | 460 (68%) | | 5,238 (67%) | 467 (64%) | | 67,229 (68%) | 2,044 (65%) | | 69,273 (68%) |
| Black or African American | 8,330 (19%) | 209 (21%) | | 4,601 (18%) | 155 (21%) | | 3,964 (18%) | 142 (21%) | | 1,416 (18%) | 154 (21%) | | 18,311 (19%) | 660 (21%) | | 18,971 (19%) |
| Other* | 4,305 (10%) | 111 (11%) | | 2,598 (10%) | 62 (8.3%) | | 2,550 (11%) | 66 (9.7%) | | 918 (12%) | 84 (12%) | | 10,371 (11%) | 323 (10%) | | 10,694 (11%) |
| Unknown/declined | 974 (2.3%) | 40 (4.1%) | | 573 (2.3%) | 28 (3.8%) | | 639 (2.9%) | 13 (1.9%) | | 228 (2.9%) | 21 (2.9%) | | 2,414 (2.5%) | 102 (3.3%) | | 2,516 (2.5%) |
| **Ethnicity** | | | <0.001 | | | <0.001 | | | 0.068 | | | 0.1 | | | <0.001 | |
| Hispanic or Latino | 7,051 (16%) | 323 (33%) | | 4,015 (16%) | 156 (21%) | | 3,966 (18%) | 140 (21%) | | 1,513 (19%) | 160 (22%) | | 16,545 (17%) | 779 (25%) | | 17,324 (17%) |
| Not Hispanic or Latino/unknown | 35,982 (84%) | 655 (67%) | | 21,153 (84%) | 588 (79%) | | 18,358 (82%) | 541 (79%) | | 6,287 (81%) | 566 (78%) | | 81,780 (83%) | 2,350 (75%) | | 84,130 (83%) |
| **Payor** | | | <0.001 | | | <0.001 | | | 0.001 | | | <0.001 | | | | |
| Private | 24,759 (58%) | 412 (42%) | | 14,890 (59%) | 380 (51%) | | 13,016 (58%) | 351 (52%) | | 4,454 (57%) | 349 (48%) | | 57,119 (58%) | 1,492 (48%) | <0.001 | 58,611 (58%) |
| Public | 17,512 (41%) | 539 (55%) | | 9,934 (39%) | 353 (47%) | | 8,914 (40%) | 316 (46%) | | 3,193 (41%) | 361 (50%) | | 39,553 (40%) | 1,569 (50%) | | 41,122 (41%) |
| Uninsured/self pay | 685 (1.6%) | 26 (2.7%) | | 317 (1.3%) | 11 (1.5%) | | 362 (1.6%) | 11 (1.6%) | | 144 (1.8%) | 15 (2.1%) | | 1,508 (1.5%) | 63 (2.0%) | | 1,571 (1.5%) |
| Other/unknown | 77 (0.2%) | 1 (0.1%) | | 27 (0.1%) | 0 (0%) | | 32 (0.1%) | 3 (0.4%) | | 9 (0.1%) | 1 (0.1%) | | 145 (0.1%) | 5 (0.2%) | | 150 (0.1%) |
| **Obesity** | 5,579 (13%) | 158 (16%) | 0.004 | 3,733 (15%) | 131 (18%) | 0.041 | 2,920 (13%) | 91 (13%) | 0.9 | 888 (11%) | 99 (14%) | 0.08 | 13,121 (13%) | 479 (15%) | 0.002 | 13,600 (13%) |
| **Asthma** | 2,397 (5.6%) | 72 (7.4%) | 0.019 | 1,512 (6.0%) | 51 (6.9%) | 0.4 | 1,332 (6.0%) | 45 (6.6%) | 0.5 | 483 (6.2%) | 52 (7.2%) | 0.3 | 5,724 (5.8%) | 220 (7.0%) | 0.005 | 5,944 (5.9%) |
| **Diabetes** | 4,388 (10%) | 143 (15%) | <0.001 | 2,633 (10%) | 95 (13%) | 0.05 | 2,377 (11%) | 71 (10%) | >0.9 | 823 (11%) | 75 (10%) | >0.9 | 10,221 (10%) | 384 (12%) | <0.001 | 10,605 (10%) |
| **Anemia** | 3,777 (8.8%) | 96 (9.8%) | 0.3 | 2,306 (9.2%) | 69 (9.3%) | >0.9 | 2,279 (10%) | 86 (13%) | 0.047 | 795 (10%) | 94 (13%) | 0.024 | 9,157 (9.3%) | 345 (11%) | 0.001 | 9,502 (9.4%) |
| **Preterm Delivery less than 37 weeks gestation** | 4,790 (11%) | 137 (14%) | 0.006 | 3,016 (12%) | 106 (14%) | 0.07 | 2,384 (11%) | 138 (20%) | <0.001 | 848 (11%) | 109 (15%) | <0.001 | 11,038 (11%) | 490 (16%) | <0.001 | 11,528 (11%) |
| **Patients with Severe Maternal Morbidities** | 690 (1.6%) | 44 (4.5%) | <0.001 | 431 (1.7%) | 35 (4.7%) | <0.001 | 343 (1.5%) | 70 (10%) | <0.001 | 109 (1.4%) | 21 (2.9%) | 0.003 | 1,573 (1.6%) | 170 (5.4%) | <0.001 | 1,743 (1.7%) |

Statistics presented: n (%); Median (IQR)

*American Indian or Alaska Native, Asian, Hispanic

© 2022 Mupanomunda M et al. *JAMA Network Open.*

**eFigure.** Standardized Mean Differences Between Groups With and Without SARS-CoV-2 Infection Before and After Propensity Matching

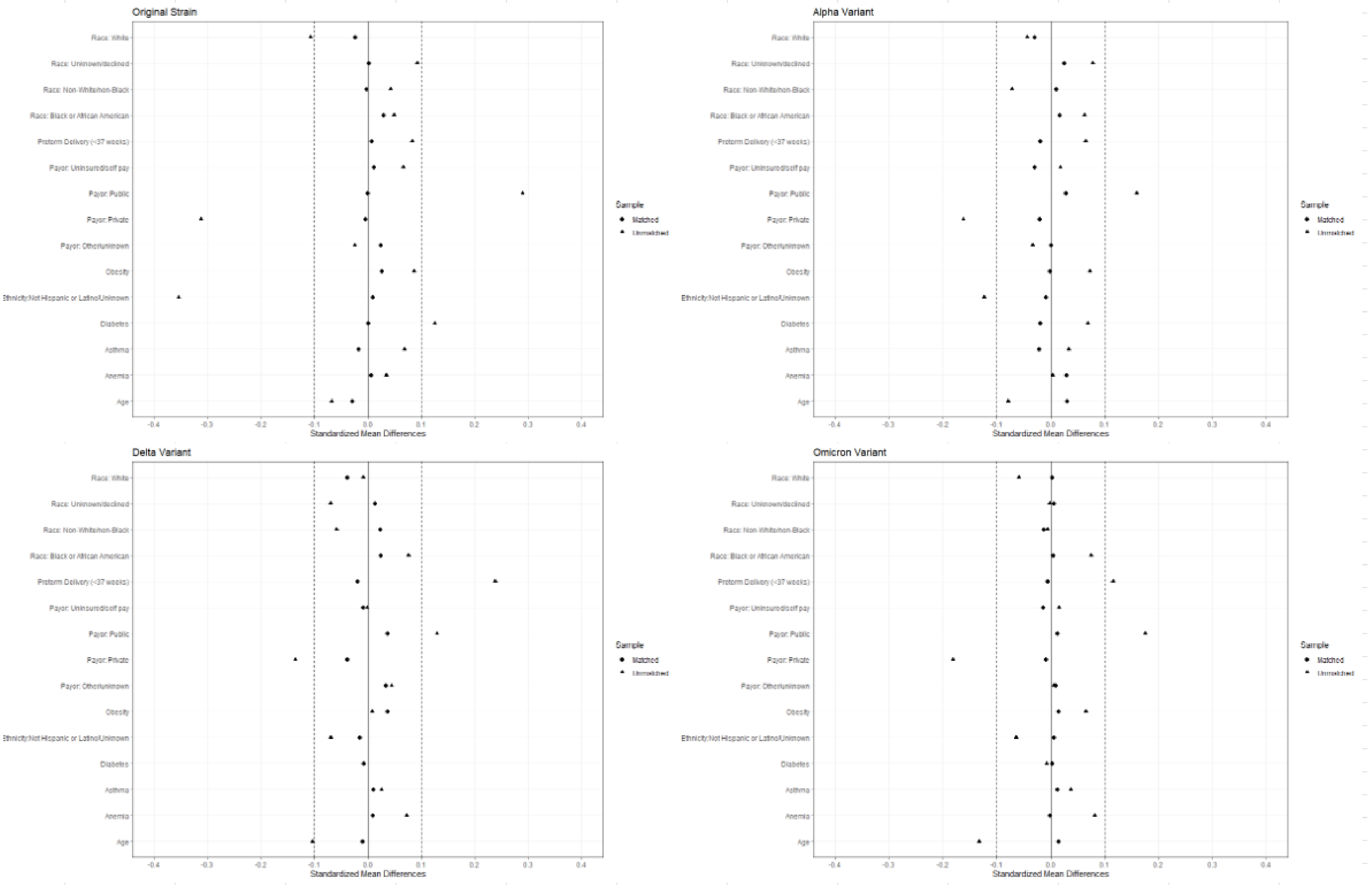


© 2022 Mupanomunda M et al. *JAMA Network Open.*

## eTable 2. Distribution of Individual SMM, by Variant

| Complication (as defined by US CDC) | Original Strain | | Alpha Variant | | Delta Variant | | Omicron Variant | |
|---|---|---|---|---|---|---|---|---|
| | Unknown/Test-Negative N = 3,906 | Test-Positive N = 978 | Unknown/Test-Negative N = 2,974 | Test-Positive N = 744 | Unknown/Test-Negative N = 2,724 | Test-Positive N = 681 | Unknown/Test-Negative N = 2,900 | Test-Positive N = 726 |
| **Acute myocardial infarction** | 0 (0%) | 0 (0%) | 1 (<0.1%) | 1 (0.1%) | 0 (0%) | 0 (0%) | 0 (0%) | 0 (0%) |
| **Aneurysm** | 0 (0%) | 0 (0%) | 0 (0%) | 0 (0%) | 0 (0%) | 0 (0%) | 0 (0%) | 0 (0%) |
| **Acute renal failure** | 1 (<0.1%) | 9 (0.9%) | 5 (0.2%) | 6 (0.8%) | 2 (<0.1%) | 8 (1.2%) | 5 (0.2%) | 1 (0.1%) |
| **Acute respiratory distress syndrome** | 3 (<0.1%) | 18 (1.8%) | 4 (0.1%) | 19 (2.6%) | 4 (0.1%) | 44 (6.5%) | 2 (<0.1%) | 7 (1.0%) |
| **Amniotic fluid embolism** | 0 (0%) | 0 (0%) | 0 (0%) | 1 (0.1%) | 0 (0%) | 0 (0%) | 1 (<0.1%) | 0 (0%) |
| **Cardiac arrest/ventricular fibrillation** | 0 (0%) | 0 (0%) | 0 (0%) | 1 (0.1%) | 0 (0%) | 2 (0.3%) | 0 (0%) | 0 (0%) |
| **Conversion of cardiac rhythm** | 0 (0%) | 0 (0%) | 0 (0%) | 1 (0.1%) | 0 (0%) | 0 (0%) | 1 (<0.1%) | 0 (0%) |
| **Disseminated intravascular coagulation** | 4 (0.1%) | 2 (0.2%) | 3 (0.1%) | 1 (0.1%) | 4 (0.1%) | 9 (1.3%) | 6 (0.2%) | 3 (0.4%) |
| **Eclampsia** | 5 (0.1%) | 2 (0.2%) | 5 (0.2%) | 0 (0%) | 2 (<0.1%) | 0 (0%) | 1 (<0.1%) | 2 (0.3%) |
| **Heart failure/arrest during surgery or procedure** | 0 (0%) | 0 (0%) | 0 (0%) | 0 (0%) | 0 (0%) | 0 (0%) | 0 (0%) | 0 (0%) |
| **Purerpural cerebrovascular disorders** | 1 (<0.1%) | 3 (0.3%) | 2 (<0.1%) | 0 (0%) | 0 (0%) | 4 (0.6%) | 1 (<0.1%) | 0 (0%) |
| **Pulmonary edema or acute heart failure** | 2 (<0.1%) | 1 (0.1%) | 5 (0.2%) | 3 (0.4%) | 1 (<0.1%) | 1 (0.1%) | 3 (0.1%) | 0 (0%) |
| **Severe anesthesia complications** | 0 (0%) | 0 (0%) | 0 (0%) | 0 (0%) | 0 (0%) | 0 (0%) | 0 (0%) | 0 (0%) |
| **Sepsis** | 5 (0.1%) | 11 (1.1%) | 1 (<0.1%) | 6 (0.8%) | 4 (0.1%) | 7 (1.0%) | 2 (<0.1%) | 2 (0.3%) |
| **Shock** | 5 (0.1%) | 3 (0.3%) | 2 (<0.1%) | 2 (0.3%) | 1 (<0.1%) | 6 (0.9%) | 0 (0%) | 1 (0.1%) |
| **Sickle cell disease with crisis** | 2 (<0.1%) | 0 (0%) | 0 (0%) | 0 (0%) | 0 (0%) | 0 (0%) | 0 (0%) | 0 (0%) |
| **Air and thrombotic embolism** | 0 (0%) | 1 (0.1%) | 0 (0%) | 0 (0%) | 0 (0%) | 1 (0.1%) | 0 (0%) | 0 (0%) |
| **Blood products transfusion** | 40 (1.0%) | 15 (1.5%) | 36 (1.2%) | 12 (1.6%) | 29 (1.1%) | 25 (3.7%) | 36 (1.2%) | 12 (1.7%) |
| **Hysterectomy** | 8 (0.2%) | 1 (0.1%) | 2 (<0.1%) | 3 (0.4%) | 2 (<0.1%) | 0 (0%) | 2 (<0.1%) | 0 (0%) |
| **Temporary tracheostomy** | 0 (0%) | 1 (0.1%) | 0 (0%) | 0 (0%) | 0 (0%) | 2 (0.3%) | 0 (0%) | 1 (0.1%) |
| **Ventilation** | 4 (0.1%) | 7 (0.7%) | 3 (0.1%) | 4 (0.5%) | 2 (<0.1%) | 15 (2.2%) | 3 (0.1%) | 3 (0.4%) |
| **Any severe maternal morbidity event** | 66 (1.7%) | 44 (4.5%) | 55 (1.8%) | 35 (4.7%) | 40 (1.5%) | 70 (10%) | 53 (1.8%) | 21 (2.9%) |
| **Any respiratory maternal morbidity event** | 6 (0.2%) | 18 (1.8%) | 5 (0.2%) | 19 (2.6%) | 5 (0.2%) | 46 (6.8%) | 4 (0.1%) | 7 (1.0%) |
| **Any non-respiratory material morbidity event** | 64 (1.6%) | 34 (3.5%) | 54 (1.8%) | 26 (3.5%) | 38 (1.4%) | 42 (6.2%) | 53 (1.8%) | 16 (2.2%) |
| **Any non-transfusion maternal morbidity event** | 32 (0.8%) | 33 (3.4%) | 22 (0.7%) | 28 (3.8%) | 17 (0.6%) | 58 (8.5%) | 18 (0.6%) | 13 (1.8%) |

© 2022 Mupanomunda M et al. *JAMA Network Open.*

## eTable 3. Baseline Characteristics Comparing Populations With Documented SARS-CoV-2 Testing After Propensity Matching

| Characteristic | Original Strain | | Alpha Variant | | Delta Variant | | Omicron Variant | |
|---|---|---|---|---|---|---|---|---|
| | Test-Positive N = 978 | Test-Negative N = 3,912 | Test-Positive N = 744 | Test-Negative N = 2,976 | Test-Positive N = 678 | Test-Negative N = 2,712 | Test-Positive N = 724 | Test-Negative N = 2,434 |
| **Age** | 29.5 (24.8, 33.5) | 29.2 (24.7, 33.4) | 29.0 (24.4, 33.5) | 29.1 (24.6, 33.5) | 29.1 (25.0, 33.1) | 29.2 (24.9, 33.1) | 28.6 (24.6, 32.8) | 29.8 (25.2, 33.9) |
| **Race** | | | | | | | | |
| White | 618 (63%) | 2,517 (64%) | 499 (67%) | 2,018 (68%) | 458 (68%) | 1,834 (68%) | 466 (64%) | 1,586 (65%) |
| Black or African American | 209 (21%) | 800 (20%) | 155 (21%) | 621 (21%) | 142 (21%) | 564 (21%) | 153 (21%) | 490 (20%) |
| Other* | 111 (11%) | 445 (11%) | 62 (8.3%) | 230 (7.7%) | 66 (9.7%) | 253 (9.3%) | 84 (12%) | 292 (12%) |
| Unknown/declined | 40 (4.1%) | 150 (3.8%) | 28 (3.8%) | 107 (3.6%) | 12 (1.8%) | 61 (2.2%) | 21 (2.9%) | 66 (2.7%) |
| **Ethnicity** | | | | | | | | |
| Hispanic or Latino | 323 (33%) | 1,320 (34%) | 156 (21%) | 625 (21%) | 139 (21%) | 553 (20%) | 160 (22%) | 648 (27%) |
| Not Hispanic or Latino/unknown | 655 (67%) | 2,592 (66%) | 588 (79%) | 2,351 (79%) | 539 (79%) | 2,159 (80%) | 564 (78%) | 1,786 (73%) |
| **Payor** | | | | | | | | |
| Private | 412 (42%) | 1,651 (42%) | 380 (51%) | 1,521 (51%) | 351 (52%) | 1,398 (52%) | 349 (48%) | 1,241 (51%) |
| Public | 539 (55%) | 2,150 (55%) | 353 (47%) | 1,408 (47%) | 316 (47%) | 1,274 (47%) | 361 (50%) | 1,161 (48%) |
| Uninsured/self pay | 26 (2.7%) | 106 (2.7%) | 11 (1.5%) | 47 (1.6%) | 11 (1.6%) | 40 (1.5%) | 14 (1.9%) | 32 (1.3%) |
| Other/unknown | 1 (0.1%) | 5 (0.1%) | 0 (0%) | 0 (0%) | 0 (0%) | 0 (0%) | 0 (0%) | 0 (0%) |
| **Obesity** | 158 (16%) | 639 (16%) | 131 (18%) | 498 (17%) | 90 (13%) | 357 (13%) | 98 (14%) | 319 (13%) |
| **Asthma** | 72 (7.4%) | 269 (6.9%) | 51 (6.9%) | 210 (7.1%) | 45 (6.6%) | 174 (6.4%) | 51 (7.0%) | 184 (7.6%) |
| **Diabetes** | 143 (15%) | 524 (13%) | 95 (13%) | 397 (13%) | 71 (10%) | 285 (11%) | 74 (10%) | 258 (11%) |
| **Anemia** | 96 (9.8%) | 398 (10%) | 69 (9.3%) | 289 (9.7%) | 86 (13%) | 343 (13%) | 94 (13%) | 288 (12%) |
| **Preterm Delivery less than 37 weeks gestation** | 137 (14%) | 553 (14%) | 106 (14%) | 450 (15%) | 138 (20%) | 547 (20%) | 107 (15%) | 291 (12%) |
| **Patients with Severe Maternal Morbidities** | 44 (4.5%) | 81 (2.1%) | 35 (4.7%) | 59 (2.0%) | 70 (10%) | 61 (2.2%) | 21 (2.9%) | 41 (1.7%) |

Statistics presented: n (%); Median (IQR)

*American Indian or Alaska Native, Asian, Hispanic

© 2022 Mupanomunda M et al. *JAMA Network Open.*

## eTable 4. Association of SARS-CoV-2 Infection With Any SMM, Nonrespiratory SMM, Respiratory SMM, and Nontransfusion SMM

| | Original Strain | | | Alpha Variant | | | Delta Variant | | | Omicron Variant | | |
|---|---|---|---|---|---|---|---|---|---|---|---|---|
| **Any SMM** | **Test-Positive** | **Test-Negative** | **Odds Ratio (95% CI)** | **Test-Positive** | **Test-Negative** | **Odds Ratio (95% CI)** | **Test-Positive** | **Test-Negative** | **Odds Ratio (95% CI)** | **Test-Positive** | **Test-Negative** | **Odds Ratio (95% CI)** |
| SMM | 44 | 81 | 2.23 (1.52, 3.22) | 35 | 59 | 2.44 (1.58,3.72) | 70 | 61 | 5.0 (3.51, 7.15) | 21 | 41 | 1.74 (1.01, 2.94) |
| No SMM | 934 | 3831 | | 709 | 2917 | | 608 | 2651 | | 703 | 2393 | |
| **Non-Respiratory SMM** | | | | | | | | | | | | |
| SMM | 34 | 80 | 1.73 (1.13, 2.57) | 26 | 57 | 1.85 (1.14, 2.94) | 42 | 61 | 2.87 (1.91, 4.28) | 16 | 41 | 1.31 (0.72, 2.32) |
| No SMM | 944 | 3832 | | 718 | 2919 | | 636 | 2651 | | 708 | 2393 | |
| **Respiratory SMM** | | | | | | | | | | | | |
| SMM | 18 | 9 | 8.13 (3.73, 19.03) | 19 | 9 | 8.64 (4.0, 20.13) | 46 | 9 | 21.86 (11.17, 47.95) | 7 | 6 | 3.95 (1.31, 12.31 |
| No SMM | 960 | 3903 | | 725 | 2967 | | 632 | 2703 | | 717 | 2428 | |
| **Non-Transfusion SMM** | | | | | | | | | | | | |
| SMM | 33 | 43 | 3.14 (1.97, 4.96) | 28 | 33 | 3.49 (2.08, 5.80) | 58 | 34 | 21.86 (11.17, 47.95) | 13 | 22 | 2.00 (0.97, 3.95) |
| No SMM | 945 | 3869 | | 716 | 2943 | | 620 | 2678 | | 711 | 2412 | |

© 2022 Mupanomunda M et al. *JAMA Network Open.*
